# Differential Responses of LINE-1 in the Dentate Gyrus, Striatum and Prefrontal Cortex to Chronic Neurotoxic Methamphetamine: A Study in Rat Brain

**DOI:** 10.3390/genes11040364

**Published:** 2020-03-28

**Authors:** Anna Moszczynska

**Affiliations:** Department of Pharmaceutical Sciences/College of Pharmacy and Health Sciences, Wayne State University, Detroit, MI 48201, USA; amosz@wayne.edu

**Keywords:** retrotransposon LINE-1, chronic methamphetamine, neurotoxicity, dentate gyrus, striatum, prefrontal cortex

## Abstract

Methamphetamine (METH) is a widely abused psychostimulant with the potential to cause a broad range of severe cognitive deficits as well as neurobehavioral abnormalities when abused chronically, particularly at high doses. Cognitive deficits are related to METH neurotoxicity in the striatum and hippocampus. The activation of transposable Long INterspersed Nuclear Element 1 (LINE-1) is associated with several neurological diseases and drug abuse, but there are very limited data regarding the effects of high-dose METH on the activity of LINE-1 in the adult brain. Using real-time quantitative PCR, the present study demonstrates that the chronic administration of neurotoxic METH doses results in the increased expression of LINE-1-encoded Open Reading Frame 1 (ORF-1) in rat striatum shortly after the last dose of the drug and decreased ORF-1 expression during METH withdrawal, with dentate gyrus potentially developing “tolerance” to these METH effects. LINE-1 activation may be a new factor mediating the neurotoxic effects of chronic METH in the striatum and, therefore, a new drug target against METH-induced psychomotor impairments in chronic METH users.

## 1. Introduction

Methamphetamine (METH) is a potent central nervous system (CNS) psychostimulant, the abuse of which remains a major public health concern worldwide. According to the SAMHSA (Substance Abuse and Mental Health Services Administration) 2018 report, there are more than 700,000 current users of METH in the United States. Alarmingly, deaths from METH overdose doubled between 2013 and 2017 and keep rising [1,2]. METH abuse causes a broad range of cognitive deficits [3], as well as neurobehavioral abnormalities [4]. Furthermore, chronic exposure to METH early in life increases the risk of developing Parkinson’s disease later on [5,6,7]. There is no FDA-approved medication for METH use disorder or specific medication that counteracts the damaging effects of METH on the adult brain [8,9]. Consequently, new drug targets are needed, particularly for heavy METH users, who suffer the most from METH abuse-related neuropsychological problems [10,11,12], are less likely to seek treatment than moderate METH users [13], and are at high risk of dying from a METH overdose [14].

METH effects on the brain are related to METH neurotoxicity, which is manifested by long-term decreases in dopaminergic and serotonergic markers in the brain, particularly in the nigrostriatal dopamine pathway [15,16,17,18,19]. The deficits in dopaminergic markers in the striatum correlate with cognitive decline and poor psychomotor functioning in abstinent METH users [20]. In contrast to striatal monoaminergic terminals, striatal neurons and interneurons in the brains of rats and humans appear not to degenerate, or show deficits in their neurotransmitters, after the administration of neurotoxic METH doses (but see mice studies [21,22]). Their function is impaired, however, due to oxidative stress and apoptotic events, including DNA oxidation and breakage [23,24,25,26]. In the hippocampus, particularly when administered at high doses, METH dysregulates neurogenesis and induces apoptosis, which is often followed by the death of pyramidal neurons and granular cells, which may contribute to the decreased hippocampal volume observed in experimental animals and humans [27,28,29,30,31,32,33,34]. These molecular events are thought to underlie a variety of cognitive impairments observed in chronic human METH users [3] and decreased spatial memory in rodents [35,36]. The prefrontal cortex is also affected by METH neurotoxicity [31,33,37], which results in impairment of executive functions in METH addicts [38].

Transposable elements are repetitive DNA sequences which have been implicated in substance use disorder [39,40,41] and, therefore, are potential drug targets in this disorder. LINE-1 belongs to the family of Long INterspersed Elements (LINEs) and is the most abundant, most active, and most intensely studied of the autonomous repetitive DNA elements [42]. LINE-1 has been implicated in several cancers [43] and in several neurodegenerative diseases [44]. LINE-1 has also been implicated in addiction. Thus, increased LINE-1 expression has been found in the mouse nucleus accumbens, after chronic exposure to psychostimulant cocaine [39], and in cultured DAergic PC12 cells chronically treated with morphine [41]. In humans, increased LINE-1 expression has been found in several brain areas of alcoholics [40]. Our group has shown that METH overdoses increase LINE-1 expression in rat neurogenic zones (subgranular zone (SGZ) of the dentate gyrus and subventricular zone (SVZ)) [45]. Altogether, these results suggest a common pathway of LINE-1 induction by drugs of abuse. This common pathway likely involves oxidative stress and apoptosis [41,46].

The goal of the present study was to determine whether the chronic administration of neurotoxic METH doses had similar effects on LINE-1 activity in the dentate gyrus and dorsal striatum as did METH overdose, in the rat. We have chosen this species because the profile of METH neurotoxicity is similar between rats and humans [47] and because LINE-1 is highly conserved in human and rodent DNA [48,49]. The LINE-1 element consists of the promoter-containing 5′ untranslated region (5′ UTR), two open reading frames, ORF-1 and ORF-2, that encode an RNA-binding protein and a protein with reverse transcriptase and endonuclease activity, respectively, and a 3′ untranslated region (3′ UTR), with a polyA tail [50]. Both ORF proteins are required for the retrotransposition of LINE-1 and non-autonomous retrotransposons. The proteins and LINE-1RNA assemble together to form ribonucleoprotein (RNP) complexes in the cytoplasm, which later move to the nucleus for reintegration into the host genome. ORF-1 protein (ORF-1p) is a nucleic acid-binding protein that lacks sequence similarity with any other known protein and its function in the brain is still unclear. It is known that ORF-1p can act as an RNA chaperone to LINE-1 RNA and other RNAs (reviewed in [51]). In addition to binding RNA, ORF-1p binds single-stranded DNA. ORF-1p is distinctive in localizing to stress granules [52], which are discrete cytoplasmic aggregates that can be induced by a range of stress conditions. In neurons, stress granules contain components required for synaptic plasticity and local protein translation [53]. ORF-2p has been shown to have a cytotoxic potential—it can break DNA via its endonuclease activity and induce apoptosis [54,55].

LINE-1 activation is often induced by hypomethylation of its promoter region [56,57]. Novel LINE-1 copies can be generated by retrotransposition that involves target-primed reverse transcription [58]. To assess LINE-1 activity after chronic METH administration, we measured ORF-1 mRNA, ORF-1 genomic DNA (gDNA) copy number and LINE-1 promoter methylation. The expression of ORF-2p was examined in the beginning and at the end of chronic METH treatment.

## 2. Materials and Methods 

### 2.1. Animals

The animals used in this investigation were young adult (~2-month-old) male Sprague–Dawley outbred rats, purchased at Harlan Laboratories (currently Envigo, Madison, WI, USA). We employed only male rats in order to compare the data from this study to the data from our already published study in binge METH-exposed male rats [45]. Binge METH (overdose) and chronic METH studies were conducted over approximately 2 years and involved multiple cohorts. The total number of rats that participated in the study was *N* = 90. Some rats died due to toxic effects of METH; however, the mortality rate was low (~6%). Some rats were removed from the study due to extensive weight loss. Upon arrival, the rats weighed 250–300 g. They were pair-housed under a 12 h light/dark cycle in a temperature-controlled (20–22 °C) and humidity-controlled room throughout the study. They had food and water available *ad libitum*. The animals acclimatized for 7 days before the start of the study. All animal procedures were conducted between 7:00 A.M. and 7:00 P.M., in strict accordance with the National Institutes of Health (NIH) Guide for the Care and Use of Laboratory Animals and were approved by the Institutional Animal Care and Use Committee (IACUC) at Wayne State University (animal protocol #16-03-067). The description of animal procedures meets the ARRIVE recommended guidelines described by The National Centre for the Replacement, Refinement and Reduction of Animals in Research [59]. 

### 2.2. Administration of Methamphetamine

**Figure 1:**
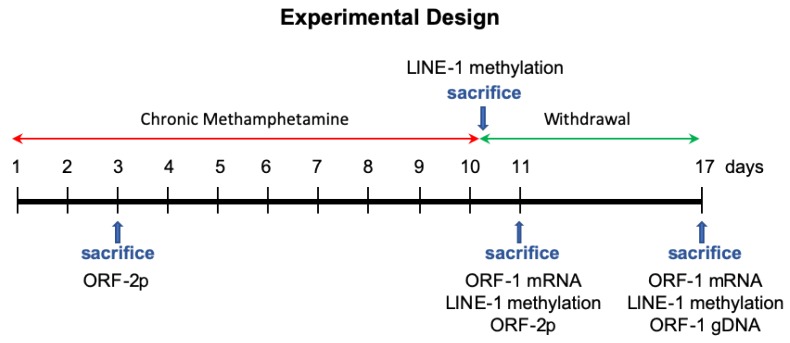
Methamphetamine (METH) was administered to young adult male rats for 10 days at the dose of 20 mg/kg/day. The control rats received saline. The animals were sacrificed at one of the indicated times (blue arrows). Dentate gyrus and striatum were assessed for the levels of ORF-2 protein (ORF-2p), ORF-1 mRNA, ORF-1 gDNA copy number and/or LINE-1 methylation.

METH metabolism in rats is much faster than in humans. Consequently, the plasma METH half-life is 10–12 h in humans vs. 60–70 min in rats. To achieve plasma METH levels in rats that are close to those in humans, higher METH doses have to be administered and/or interval between METH injections shortened in studies employing rats. To simulate heavy METH abuse in humans, rats were administered daily doses of (+)-METH hydrochloride (20 mg/kg free base) (Sigma-Aldrich, St. Louis, MO, USA) for 10 days via intraperitoneal (i.p.) injections. Control rats received saline (1 mL/kg) at the same time. This METH dosing induces neurotoxicity [19]. METH neurotoxicity is associated with hyperthermia, which peaks at approximately 1 h after each injection. Therefore, core body temperatures were measured with a rectal probe digital thermometer (Thermalert TH-8; Physitemp Instruments, Clifton, NJ, USA), before the beginning of the treatment (baseline temperatures) and at 1 h after each METH or saline injection. Rats were sacrificed by decapitation on the 3rd day of chronic METH treatment (for ORF-2p analysis), or after the treatment: at 1 h (for LINE-1 methylation analysis), 24 h or 7 days (for LINE-1 methylation, expression and/or gDNA copy# analysis) after the last injection of the drug or saline. The experimental design is presented in Figure 1.

### 2.3. Tissue Collection

The brains were removed, dissected out into discrete brain areas (striatum, dentate gyrus, prefrontal cortex, and cerebellum) and stored at −80 °C until assayed. The SVZ was dissected out together with the striatum, whereas the SGZ was dissected out together with the dentate gyrus, using punch sampling technique. Muscle tissue was also collected and stored at −80 °C until analysis. 

### 2.4. Real-Time Polymerase Chain Reaction and Pyrosequencing

Real-time quantitative polymerase chain reaction (qPCR) was employed to measure the levels of ORF-1 mRNA and ORF-1 gDNA copy number in the dissected brain areas from METH- or saline-treated rats that were sacrificed on the 1st or 7th day of METH withdrawal (Figure 1). A pyrosequencing technique was employed to determine the methylation of the first ten CpG sites within the LINE-1 promoter region in rats sacrificed at 1 h, 24 h or 7 days after METH (Figure 1). Glyceraldehyde-3-phosphate dehydrogenase (GADPH) was used as a reference gene. The analyses were conducted at EpigenDx Inc. (Hopkinton, MA, USA). The details of these analyses can be found in a previously published manuscript [45]. The data are expressed as LINE-1/GADPH ratios normalized to saline controls (mean ± SEM). This approach normalizes the differences between runs. 

### 2.5. Immunohistochemistry

For immunohistochemistry, another cohort of young adult male rats was administered METH or saline, as described above. These rats were sacrificed by decapitation on the 3rd day of chronic METH treatment (Figure 1). The brains were dissected out, fixed in 4% paraformaldehyde, cryoprotected by incubation with 20% and 30% glycerol, snap-frozen, and stored at −80 °C. Every other of the coronal sections (20 μm, 3/rat) from the striatum/SVZ (1.18–0.26 from Bregma) and the dentate gyrus/SGZ (−3.12 to −4.68 from Bregma) was collected for analysis. Sections were pretreated with 1× citrate buffer for 40 min at 70 °C, then allowed to cool to room temperature, before being blocked in a blocking buffer (phosphate-buffered saline (PBS), 0.1% Triton X-100, and 5% bovine serum albumin (BSA)) for 1 h at room temperature. These sections were then incubated overnight at 4 °C with a chicken anti-ORF-2p (1:200, Rockland Immunochemicals Inc., Limerick, PA, USA) primary antibody. Of note, we did not employ an ORF-1p antibody in the present investigation, because this antibody did not work in our hands. The coronal sections were next incubated with anti-chicken secondary antibody (Alexa-488, 1:1000, 3 h at room temperature). The nuclei were labeled with DRAQ5 dye (Life Technologies, Carlsbad, CA, USA). The sections were mounted on slides using Fluoromount mounting medium (Sigma-Aldrich, St. Louis, MO, USA). Immunofluorescence on each slice was measured under the same settings, using Leica TCS SPE-II confocal microscope with spectral detector and Application Suite Advanced Fluorescence software. All data were averaged first per slice and then per rat, and normalized to saline-treated controls after each experiment. This approach normalized differences across the experimental days and allowed for standardization across the treatment groups.

### 2.6. Statistical Analysis

The comparisons made in the study were pre-planned comparisons. We established a priori the dentate gyrus/SGZ and striatum/SVZ as potentially affected brain regions in chronic METH-exposed rats and chose other samples based on existing knowledge regarding METH neurotoxicity and LINE-1 effects on the brain and muscle. Significant differences between the control group and METH group were determined using multiple unpaired two-tailed *t*-tests, followed by the Holm-Sidak method (*p* < 0.05 was corrected to adjust for the probability of type I errors in multiple comparisons). A consistent standard deviation was assumed for each brain area. A two-way repeated-measures Analysis of Variance (ANOVA), followed by the Sidak post hoc test, was performed on the temperature data. Correlations were determined using Pearson’s analysis. The data sets are expressed as the mean ± SEM (standard error of the mean). 

## 3. Results

### 3.1. Chronic METH Treatment Increases ORF-1 mRNA Levels in the Striatum of Adult Rat Brain

**Figure 2:**
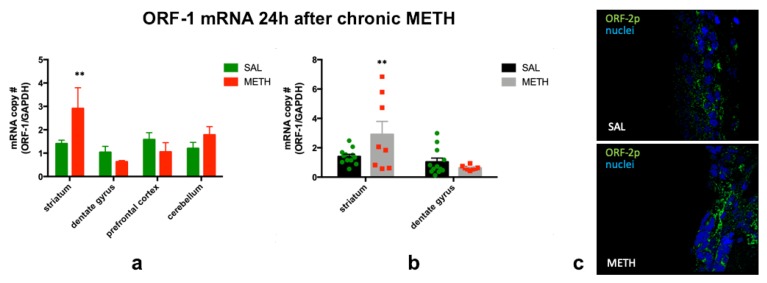
(**a**) The effect of chronic METH (20 mg/kg/d, for 10 d, i.p.) on ORF-1 mRNA levels in rat striatum, dentate gyrus, prefrontal cortex and cerebellum at 24 h after the last dose of METH or saline. (**b**) Individual mRNA values in the striatum and dentate gyrus. (**c**) Representative image of increased immunofluorescence for ORF-2p in the SVZ. Abbreviations: h, hour; METH, methamphetamine; SAL, saline; SVZ, subventricular zone. ** Statistical significance *p* < 0.01, METH vs. SAL.

We have previously determined that neurotoxic METH overdose (4 × 10 mg/kg, i.p., every 2 h) increases LINE-1 DNA transcription in the striatum/SVZ and dentate gyrus/SGZ at 24 h after METH (1.8- and 2.3-fold, respectively), but not in the prefrontal cortex, cerebellum, or control muscle [45]. Our group and others showed that chronic high-dose METH administration (dose range: 5–50 mg/kg for 4–12 days) is also neurotoxic; it causes significant depletion of dopaminergic and serotonergic markers as well as neuronal apoptosis and/or necrosis in rat striatum, prefrontal cortex, and hippocampus [17,19,27,60,61,62,63,64,65]. In this study, we employed our rat model of neurotoxicity of chronic METH [19] and assessed indices of LINE-1 activity. Statistical analysis of ORF-1 mRNA levels in the striatum, dentate gyrus, prefrontal cortex and cerebellum of rats treated with neurotoxic chronic METH or saline revealed induction of ORF-1 transcription to mRNA at 24 h after chronic METH (2-fold, *p* = 0.005, *t* = 2.93, *df* = 66, *n* = 4–13, individual *t*-tests with Holm–Sidak correction for multiple comparisons), but not in the dentate gyrus (Figure 2a). Chronic neurotoxic METH treatment produced variable responses in the striatum (high standard deviation in the METH group); however, a subgroup of METH rats showed a definitive increase in ORF-1 expression (Figure 2b). The ORF-1 mRNA levels in the prefrontal cortex and cerebellum did not differ between chronic METH-treated rats and chronic saline-treated controls (Figure 2a). Relative to induced LINE-1 mRNA production by chronic METH treatment in brain tissue, no induction by METH was detected in negative control muscle tissue (SAL: 0.066 ± 0.021; METH: 0.070 ± 0.014, *p* > 0.1, Student’s *t*-test, *t* = 0.143, *df* = 7; *n* = 4–5). To determine whether the increased LINE-1 transcription is accompanied by increased LINE-1 mRNA translation, the levels of ORF-2p protein were measured by immunohistochemistry. The ORF-2p signal was present within the SVZ and was higher in some METH-treated rats as compared to saline controls. Figure 2c shows an example of increased ORF-2p immunoreactivity in the SVZ.

### 3.2. Expression of ORF-2p is Increased on the Third Day of Chronic Methamphetamine Treatment

**Figure 3:**
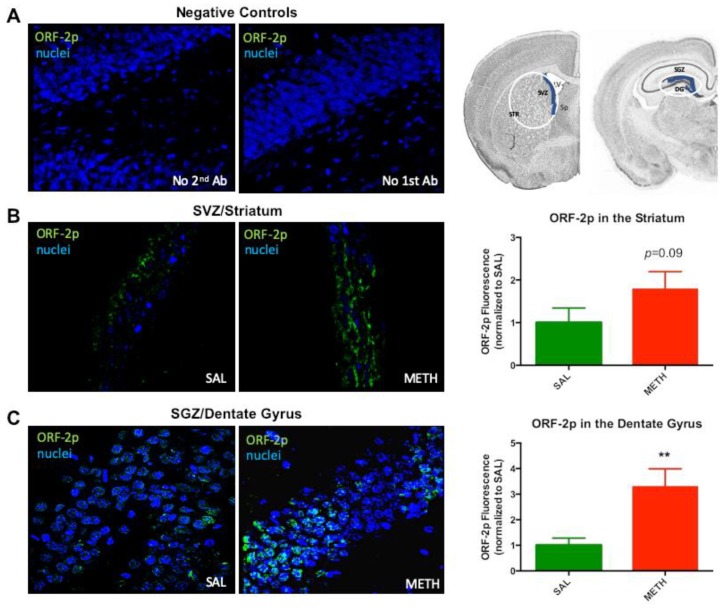
The effect of two daily 20 mg/kg METH doses on ORF-2p immunoreactivity in rat striatum and dentate gyrus at 24 h after the 2nd injection of METH or saline. (**A**) No-secondary (2nd) antibody control, no-primary (1st) antibody control and graphic presentation of dissected out tissue (white ovals), with delineated subventricular and subgranular zones (SVZ and SGZ, blue lines). (**B**) ORF-2p immunoreactivity in the SVZ. (**C**) ORF-2p immunoreactivity in the SGZ and dentate gyrus. Abbreviations: METH, methamphetamine; SAL, saline; SGZ, subgranular zone; SVZ, subventricular zone; ORF-2p, ORF-2 protein. ** Statistical significance, *p* < 0.01, METH vs. SAL.

It has been reported that oxidative stress induces increased LINE-1 expression [66]. A dose of 40 mg/kg METH administered in two doses over two days could induce less severe oxidative stress than the same total dose administered over 6 h (binge METH). Consequently, assuming oxidative stress upstream of LINE-1 activation needs to reach a threshold, LINE-1 activation may not have occurred in the dentate gyrus, thus explaining the lack of an increase in ORF-1 mRNA later on. Alternatively, METH induced an increase in ORF-1 mRNA in the beginning of chronic administration of the drug, but “tolerance” developed to this effect over time. To test this hypothesis, rats were treated with 20 mg/kg METH, or saline, per day for two days, and sacrificed 24 h later (Figure 1). Immunoreactivity of ORF-2p was found to be increased in the SVZ (1.8-fold, *p* = 0.091, *t* = 1.46, *df* = 8, *n* = 5) and dentate gyrus/SGZ (3.3-fold, *p* = 0.009, *t* = 2.99, *df* = 8, *n* = 5) at 24 h after the second METH dose as compared to saline controls (Figure 3), indicating that LINE-1 was activated in these areas in the beginning of the chronic METH administration. ORF-2p localized to the cytoplasm.

### 3.3. Chronic METH Treatment Leads to a Persistent Decrease in ORF-1 mRNA Levels in the Striatum of Adult Rat Brain

**Figure 4:**
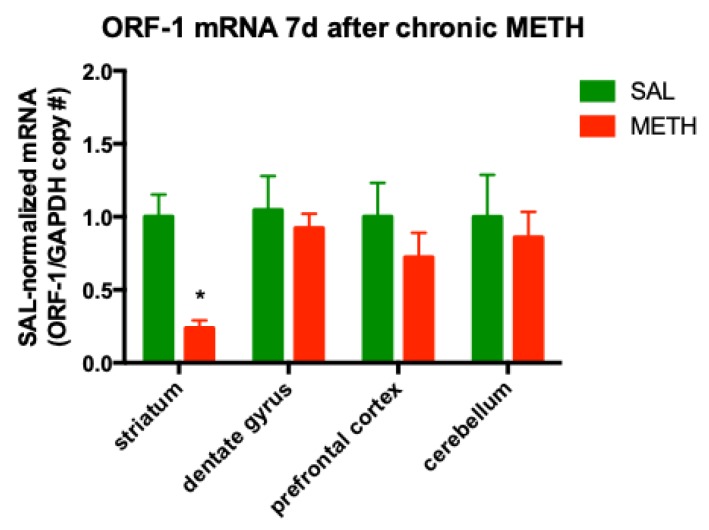
The effect of chronic METH (20 mg/kg/d, for 10 d, i.p.) on ORF-1 mRNA levels in the striatum, dentate gyrus, prefrontal cortex and cerebellum at 7 d after the last dose of METH or saline. Abbreviations: d, day; METH, methamphetamine; SAL, saline. * Statistical significance *p* < 0.05, METH vs. SAL.

At 24 h after METH treatment, the effects of the last METH injection can be mixed with the chronic effects of the drug. To determine the effects of chronic METH alone on ORF-1 expression, another cohort of rats was treated with chronic METH or chronic saline, and sacrificed on the seventh day of METH withdrawal. The striatum displayed a significant decrease in ORF-1 mRNA levels (−76%, *p* = 0.018, *t*-test corrected for four comparisons by Holm–Sidak method, *t* = 2.52, *df* = 25, *n* = 4–7), while ORF-1 mRNA levels in the dentate gyrus were not different from saline-treated controls (Figure 4). Since we previously reported that ORF-1 mRNA levels in the striatum were significantly but moderately (−57%) decreased in binge METH-treated rats, compared to saline controls at seven days after the last METH dose [45], the present finding suggested the further chronic METH-induced adaptive down-regulation of LINE-1 transcription in the striatum. Chronic neurotoxic METH did not significantly change in the ORF-1 mRNA levels in the prefrontal cortex or cerebellum (Figure 4). Likewise, no ORF-1 transcription induction by METH was detected in muscle tissue (SAL: 1.00 ± 0.08; METH: 0.98 ± 0.14, *p* > 0.1, Student’s *t*-test, *t* = 0.086, *df* = 5; *n* = 4).

### 3.4. Chronic METH Has no Significant Effect on the ORF-1 gDNA Levels in the Striatum and Dentate Gyrus of Adult Rat Brain

**Figure 5:**
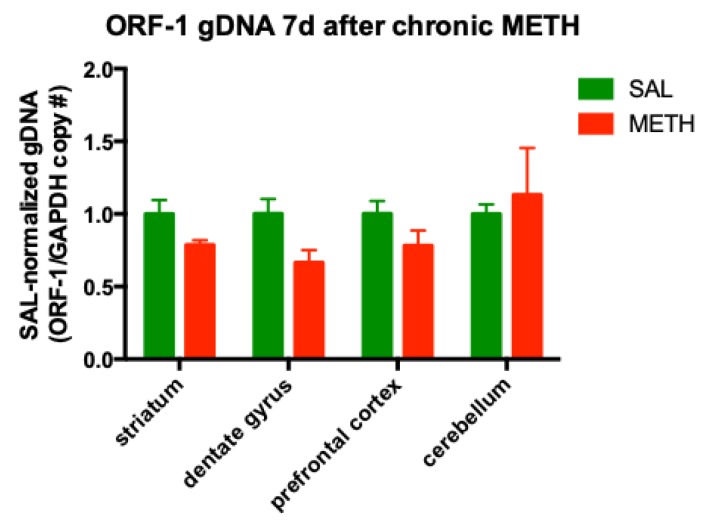
The effect of chronic METH (20 mg/kg/d, for 10 d, i.p.) on ORF-1 copy number within the genomic DNA (gDNA) in rat striatum, dentate gyrus, prefrontal cortex and cerebellum, at 7 d after the last dose of METH or saline. Abbreviations: d, day; METH, methamphetamine; SAL, saline.

The most identified function of LINE-1 RNA is working as a template for copying itself through reverse transcription, and therefore directing the paste process of integrating itself into gDNA [61]. LINE-1 undergoes retrotransposition via a copy-and-paste mechanism, and thereby increases its copy number within gDNA [62]. To assess whether the increase in LINE-1 mRNA production induced by chronic METH treatment was potentially followed by LINE-1 transposition into the gDNA, we quantified ORF-1 gDNA copy numbers in the brains of rats treated with chronic METH or saline and sacrificed after a seven-day-long withdrawal from METH. Statistical analysis of the dissected brain areas by individual *t*-tests and corrected for multiple comparisons using the Holm–Sidak method did not reveal statistically significant changes in ORF-1 gDNA copy number in the striatum or dentate gyrus (striatum: *p* = 0.291, *t* = 1.08, *df* = 53; dentate gyrus: *p* = 0.102, *t* = 1.69, *df* = 24; *n* = 4) (Figure 5). When the dentate gyrus and striatum were combined into one neurogenic group, the ORF-1 gDNA levels were significantly decreased in METH-treated rats compared to the saline controls (−28%, *p* < 0.05, Student’s *t*-test, *t* = 3.49, *df* = 6, *n* = 4). This finding suggested ORF-1-containing cell loss within these two brain regions. The prefrontal cortex and cerebellum had basal levels of ORF-1 gDNA at 7 days after chronic METH regiment (Figure 5). The levels of ORF-1 mRNA in muscle tissue also did not significantly differ between METH- and saline-treated rats (SAL: 1.00 ± 0.23; METH: 1.21 ± 0.17, *p* > 0.1, Student’s *t*-test, *t* = 0.697, *df* = 6; *n* = 4).

### 3.5. Chronic METH-Triggered Activation of LINE-1 Is Not Accompanied by Hypomethylation of LINE-1 Promoter

**Figure 6:**
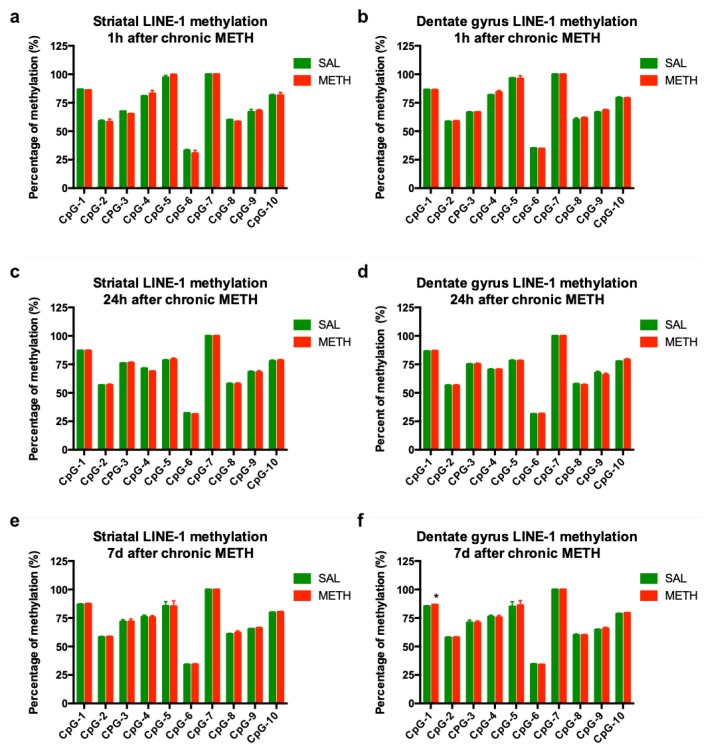
The effect of chronic METH administration (20 mg/kg/d, for 10 d, i.p.) on LINE-1 promoter methylation in the striatum (**a**,**c**,**e**) and dentate gyrus (**b**,**d**,**f**) at 1 h (**a**,**b**), 24 h (**c**,**d**), and 7 d (**e**,**f**) after the last dose of METH or saline. Abbreviations: d, day; METH, methamphetamine; SAL, saline. * Statistical significance *p* < 0.05, METH vs. SAL.

LINE-1 activation is often induced by the hypomethylation of its promoter region [56,57]. The examination of the first 10 CpG sites within the promoter region-containing 5’ UTR of LINE-1 revealed no statistically significant changes in the average methylation of these sites in the striatum or dentate gyrus of chronic METH-exposed rats relative to saline controls at 1 h or 24 h after chronic METH. Similarly, no decreases were observed at individual CpGs in the striatum or dentate gyrus (Figure 6a–d). Methylation of the LINE-1 promoter at the seven-day time point was assessed, to determine whether hypermethylation accompanied the decrease in striatal ORF-1 mRNA. No statistically significant changes were observed in the striatum (Figure 6e). A single statistically significant change found was the hypermethylation of CpG-1 site in the dentate gyrus (Figure 6f). Of note, methylation status of LINE-1 was not significantly altered by binge METH in any brain region at 1 h, 24 h or 7 d, with the exception of the dentate gyrus (−1%, 24 h after METH overdose [45]).

### 3.6. Methamphetamine-Induced Hyperthermia Does Not Contribute to LINE-1 Activation

**Figure 7:**
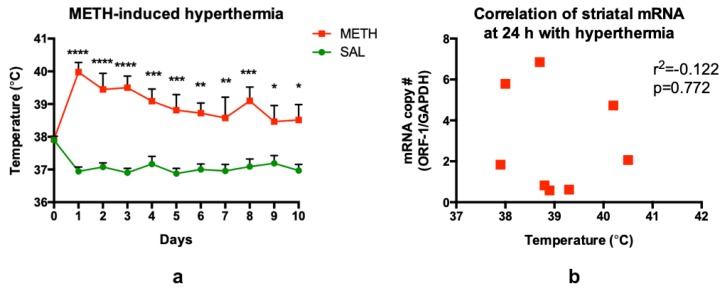
(**a**) Chronic METH administration (20 mg/kg/d, for 10 d, i.p.) induces significant core body temperature elevations, lasting throughout the treatment (10 d). (**b**) ORF-1 mRNA levels measured 24 h after METH treatment did not correlate with METH-induced hyperthermia. Abbreviations: METH, methamphetamine; SAL, saline. * *p* < 0.05, ** *p* < 0.01, *** *p* < 0.001, **** *p* < 0.0001, METH vs. SAL.

METH-induced hyperthermia is an important contributor to METH neurotoxicity [67] and, therefore, can potentially contribute to LINE-1 activation. Rat core body temperatures were recorded at 1 h after each METH or saline injection, and the data are presented in Figure 7a. As expected, METH induced statistically significant increases in core body temperatures compared to saline (38.5–39.9 °C vs. 36.9–37.9 °C). There was a significant main effect of the treatment (F_(1,14)_ = 31.0, *p* < 0.0001) and time (F_(9,126)_ = 5.24, *p* < 0.001), as well as significant treatment × time interaction (F_(9,126)_ = 2.71, *p* < 0.01), as determined by two-way ANOVA with repeated measures, followed by Sidak’s post hoc test. There was no correlation between ORF-1 24 h-mRNA levels and hyperthermia in the striatum (Pearson’s two-tailed correlation analysis, *r^2^* = −0.122, *p* = 0.772) (Figure 7b), suggesting that the increase in ORF-1 mRNA levels at 24 h was not caused by the increase in core body temperature. 

## 4. Discussion

The present study demonstrates that the systemic chronic administration of neurotoxic METH doses to young adult male rats increases striatal ORF-1 mRNA levels at 24 h after the last dose of the drug, in a LINE-1 promoter hypomethylation- and hyperthermia-independent manner, and leads to their decrease during withdrawal from the drug. The study also demonstrates that the dentate gyrus develops a “tolerance” to ORF-1 mRNA-increasing effect of METH during chronic administration of the drug and that chronic METH administration does not lead to significant increases in striatal ORF-1 DNA copy number at 7 d after the last METH injection, in either region. Lastly, the study reports no signs of METH-induced LINE-1 activation in the prefrontal cortex.

We have previously demonstrated that that high-dose binge METH (4 × 10 mg/kg, over 6 h) increases ORF-1 mRNA and ORF-2p levels within the neuronal cytoplasm of the dentate gyrus/SGZ and striatum/SVZ, at 24 h after the last METH injection [45]. A lack of increase in dentate gyrus ORF-1 mRNA observed in the current study at 24 h after chronic METH could have been due to the lack of LINE-1 activation by daily 20 mg/kg METH doses, as opposed to 40 mg/kg METH binge administered over 6 h. Against this scenario, we found increased ORF-2p levels in the dentate gyrus/SGZ and in the SVZ on the third day of chronic METH treatment. Thus, the present finding of high-dose chronic METH increasing ORF-1 mRNA in the striatum but not in the dentate gyrus at 24 h suggests that “tolerance” develops to ORF-1 mRNA-increasing effects of METH in the dentate gyrus/SGZ, but not in the striatum/SVZ, during 10 d of chronic administration of the drug. This “tolerance” may have involved effective attenuation of oxidative stress [19,68], which is upstream of LINE-1 activation [46,66,69], and/or accelerated LINE-1 RNA decay/mRNA degradation in response to METH-induced oxidative stress and apoptosis [70,71,72].

Increased LINE-1 expression (nuclear RNA) at the 24 h time point has also been found in the mouse nucleus accumbens after chronic exposure to another psychostimulant, cocaine [39]. In vitro, LINE-1 was activated upon exposure to METH, cocaine [73], morphine [41], or nicotine [74]. In human studies, signs of increased LINE-1 activity were detected in the amygdala and the superior frontal cortex of alcoholics [40], the medial prefrontal cortex of cocaine users [75] and the cell-free DNA of cigarette smokers [76]. These results suggest a common pathway of LINE-1 induction by these substances. The available data implicate an imbalance in redox status as a common pathway leading to LINE-1 activation. Thus, METH, cocaine, morphine, cigarette smoke and alcohol can all induce oxidative stress and deficit in antioxidant glutathione [19,41,77,78]. Oxidative stress, or glutathione deficiency, increase LINE-1 mRNA levels in cell culture [46,69] and in vivo [66]. Conversely, the reduction of LINE-1 activity protects neurons against oxidative stress in vitro and in vivo [66]. Furthermore, it has been reported that the inhibition of LINE-1 expression in the heart decreased ischemic damage by activation of Akt/PKB signaling [79], which is regulated by oxidative stress [80]. The notion of imbalanced redox status playing a role in LINE-1 activation is also supported by recent reports of increased LINE-1 activity in aging [81], a life stage during which antioxidant defenses decrease while oxidative stress level rises. The aforementioned studies measured LINE-1 activation by different assays (hypomethylation, retrotransposition, RNA levels et al.); increased ORF-1 expression after METH was reported by Okudaira and colleagues (ORF-1 protein levels) [73] and by our group (ORF-1 mRNA) [45].

Some tolerance does develop to METH hyperthermia (Figure 7), as well as to the neurotoxic effects of METH in the striatum, hippocampus and prefrontal cortex upon repeated administration of the drug [19,82,83], with the tolerance to METH-induced neurotoxicity shown not being due to the attenuation of METH-induced hyperthermia [82]. Our present results also suggest that mechanisms underlying “tolerance” to ORF-1 mRNA-increasing effect of METH are not mediated by hyperthermia. However, these mechanisms may also be different than mechanisms contributing to the tolerance to METH neurotoxicity at the molecular level, because tolerance to ORF-1 mRNA-increasing effect of METH was not observed in the striatum. High-dose binge METH decreased glutathione levels in rat striatum at 3 h, while chronic high-dose METH (20 mg/kg, 10 d, i.p.) did not have this effect [19], indicating tolerance to METH-induced oxidative stress and/or recovery of glutathione synthesis in this brain area during 10 d of chronic METH treatment. Consequently, even though glutathione deficit may play a role in initial LINE-1 activation, it is rather, not a factor keeping ORF-1 mRNA levels increased at 24 h after METH in the striatum/SVZ. It is plausible that ORF-1 mRNA went up higher and/or stayed up longer in the striatum/SVZ than in the dentate gyrus/SGZ, due to regional differences in LINE-1 RNA metabolism or stress granules dynamics. Stress granules store translation-dormant mRNAs that may be directed for degradation. Stress granules also contain LINE-1 RNA which can be directed to decay via interaction with MOV10 helicase [70,84]. Consequently, “tolerance” to the ORF-1 mRNA-increasing effect of METH may be due to accelerated decrease in these RNAs. Chronic METH can induce apoptosis and cell death in the hippocampus [27,85]; therefore, it is also possible that ORF-1 mRNA decreased in this brain region, due to the accelerated death of cells containing ORF-1 mRNA during chronic treatment with METH. 

The striatum, hippocampus, and prefrontal cortex, as well as SGZ and SVZ, contain dopaminergic and glutamatergic innervations [86,87,88]. High-dose METH triggers dopamine and glutamate release in the striatum and hippocampus [89,90], followed by oxidative stress, excitotoxicity, compromised antioxidant glutathione system and, finally, nerve terminal degeneration in these brain areas (striatum [19,23,24,25,26,29,91] and hippocampus [29,91,92,93,94]). What sets apart these two brain regions the most in relation to METH neurotoxicity is dopamine content. The striatum is more dopamine-rich compared to the hippocampus. Consequently, dopamine release and post-synaptic signaling is the highest in the striatum after administration of the same dose of METH. In view of our data, this implicates that “tolerance” to ORF-1 mRNA-increasing effect of METH is not mediated by dopamine, but perhaps a sustained increase in the ORF-1 mRNA is mediated. Thus, a simple explanation for our finding is that ORF-1 mRNA increased more in the striatum than in the dentate gyrus, and did not fall to the basal levels in the former by 24 h after the last METH dose. The prefrontal cortex contains about 100 times less dopamine than the striatum and, therefore, it is less affected by binge or chronic METH, due to lower DA release and related oxidative stress [47]. Moreover, glutamate release is not increased in the prefrontal cortex by repeated METH dosing [83], as it is in the striatum [95] and hippocampus [90], which may explain the lack of LINE-1 activation in this area. We have previously found that both dopamine and glutamate increase LINE-1 retrotransposition in vitro, with glutamate having a stronger effect [45]. Consequently, regional differences in LINE-1 responses to chronic METH can be due to differences in extracellular glutamate levels as well. Along these lines, regional differences have been observed between the prefrontal cortex, striatum and hippocampus in glutamatergic system responses to acute and repeated METH. For example, chronic exposure to medium-dose METH (4 mg/kg/d, i.p. for 14 d) resulted in differential changes in glutamate transporter EAAT3: a 39% decrease in the striatum, 25% decrease in the prefrontal cortex and 72% increase in the hippocampus [96]. Dopamine transporter was also differentially affected by chronic METH, with dorsal striatum showing a higher deficit than the prefrontal cortex and hippocampus showing no deficit [97]. Thus, increased glutamate uptake in the hippocampus may be responsible, at least in part, for the “tolerance” to ORF-1 mRNA-increasing effect of METH in the present study. The METH-triggered increase in glutamate is paralleled by an up-regulation in the expression of vesicular glutamate monoamine transporter 1 in the striatum [98]. Due to the fact that this transporter determines the amount of glutamate loaded into storage vesicles and consequently determines the release of glutamate [99], its increased expression may contribute to the prolonged release of glutamate in the striatum/SVZ following high-dose METH and the prolonged activation of LINE-1 in the SVZ.

Binge administration of high METH doses resulted in increased ORF-1 DNA copy number within the dentate gyrus and striatum on the seventh day of forced METH withdrawal [45]. Chronic administration of high METH doses did not lead to increases in ORF-1 DNA copy number at this time point, suggesting no retrotransposition of LINE-1 upon exposure to this METH regiment. Since we previously observed increased ORF-1 copy number 24 h after binge METH, an alternative hypothetical explanation for our current observation is initial LINE-1 retrotransposition, followed by the death of SVZ cells that underwent LINE-1 retrotransposition. Another potential interpretation of the observed results is that LINE-1 expression is induced without subsequent retrotransposition of the element. Chronic oxidative stress leads to the accumulation of stress granules [100]. Stress granules contain ORF-1p and have been suggested to regulate LINE-1 retrotransposition. Consequently, an increase in the stress granule number during chronic METH treatment could have restricted the number of LINE-1 retrotransposition events, as suggested by Goodier and colleagues [52]. Alternatively, it is plausible that DNA repair enzymes were able to prevent LINE-1 retrotransposition due to sub-threshold level of oxidative stress [66]. Finally, it is possible that METH-induced increases in ORF-1 copy number were extrachromosomal [101,102,103,104] and dissipated by the 7th day of withdrawal from METH.

Rat LINE-1 is strongly methylated at CpG-dinucleotides in most genomic copies of various somatic tissues, including the brain [105]. Compelling research data indicate that the methylation of the internal promoter located in LINE-1′s 5′ UTR determines LINE-1 transcription, especially the methylation status of the first few CpGs being identified as a sufficient and necessary factor for controlling LINE-1 promoter activity [56]. We did not detect decreases in striatal or dentate gyrus LINE-1 methylation, either at 1 h or 24 h after the last METH injection, as compared to saline-treated rats. We also did not detect the altered methylation of LINE-1 promoter at 7 d after chronic METH administration. It can be argued that the hypomethylation of the element occurred in the beginning of chronic METH treatment. Against this argument, we previously observed a lack of significant LINE-1 hypomethylation at 1 h or 24 h after binge METH administration [45]. In summary, our previous and current results, together with the finding that morphine-increased LINE-1 expression did not correlate with LINE-1 hypomethylation [41], point to mechanisms independent of cytosine methylation at the LINE-1 promoter CpG sites. Potential mechanisms regulating LINE-1 activation and transcription during METH administration include LINE-1 hydroxymethylation [106], as well as HDACs-mediated histone modifications, small interfering RNAs, small piRNAs, P1-LINE-1 RNA, DNA-editing proteins, and WTN pathway [50,105,107,108,109,110,111]. As compared to saline controls, rats that self-administered high doses of METH showed differential DNA hydroxymethylation within LINE; therefore, this mechanism is the one most likely responsible for METH-triggered LINE-1 activation. Histone-mediated LINE-1 activation is another possibility, as METH induces multiple histone modifications [112]. Changes in the LINE-1 allele methylation pattern have recently been applied to the assessment of LINE-1 methylation in chronic human METH users and detected changes in partial LINE-1 methylation [113,114]. This finding introduces a possibility of similar changes in chronic METH-exposed rats.

## 5. Conclusions

Overall, our data suggest that the chronic administration of high METH doses persistently activates LINE-1 in the SVZ and leads to the decreased transcription of LINE-1-encoded ORF-1 gene, accelerated removal of LINE-1 RNA or ORF-1 mRNA, or ORF-1-positive cell death within the SVZ during the withdrawal. Our data also suggest that some of these processes may occur earlier in the dentate gyrus/SGZ than in the SVZ. LINE-1 activation might be a new factor of mediating the neurotoxic effects of chronic METH in the striatum, which are manifested by persistent psychomotor impairments in human METH users and can lead to Parkinson’s disease. The development of “tolerance” to METH-induced increase in ORF-1 mRNA by the dentate gyrus/SGZ, as well as the lack of signs for the LINE-1 activation in the prefrontal cortex, suggests that LINE-1 in the striatum/SVZ, dentate gyrus/SVZ and the prefrontal cortex respond in a different manner to chronic METH administration. These differential responses are likely due to, at least in part, differences in the extracellular levels of dopamine and glutamate after exposure to METH, summarized in Table 1.

## Figures and Tables

**Table 1:** The effects of METH on LINE-1 activity and extracellular levels of dopamine and glutamate in the dorsal striatum, dentate gyrus, and prefrontal cortex.

Brain Area	Dorsal Striatum	Dentate Gyrus	Prefrontal Cortex
LINE-1 activity (binge METH)	⇑ ⇑ [45]	⇑ ⇑ [45]	= [45]
Extracellular DA after binge METH	⇑ ⇑ [44]	⇑ [44]	⇑ [44]
Extracellular GLU after binge METH	⇑ ⇑ [76]	⇑ [81]	= [76]
**LINE-1 activity (chronic METH)**	**⇑** **⇑**	**=**	**=**
**LINE-1 “tolerance” (chronic METH)**	**No**	**Yes**	**NA**
GLU transporter after chronic METH	⇓ ⇓ [87]	⇑ [87]	⇓ [87]
DAT transporter after chronic METH	⇓ ⇓ [88]	= [88]	⇓ [88]

⇑ ⇑: marked increase, ⇑: moderate increase, ⇓ ⇓: marked decrease, ⇓: moderate decrease, =: no change. Abbreviations: DA, dopamine; GLU, glutamate; METH, methamphetamine; NA, not applicable. **Bold** represents current studies.
